# Effects of radioiodine therapy on fertility indicators among men with differentiated thyroid cancer: A cohort study

**DOI:** 10.18502/ijrm.v21i5.13472

**Published:** 2023-05-12

**Authors:** Salman Soltani, Atena Aghaee, Seyed Rasoul Zakavi, Mahdi Mottaghi, Maryam Emadzadeh, Soheil Kasaeian Naeini

**Affiliations:** ^1^Kidney Transplantation Complications Research Center, Mashhad University of Medical Sciences, Mashhad, Iran.; ^2^Nuclear Medicine Research Center, Mashhad University of Medical Sciences, Mashhad, Iran.; ^3^Clinical Research Development Unit, Ghaem Hospital, Mashhad University of Medical Sciences, Mashhad, Iran.

**Keywords:** Follicle-stimulating hormone, Iodine-131, Male infertility, Semen analyses.

## Abstract

**Background:**

Following thyroidectomy, radioiodine therapy is the standard management of differentiated thyroid cancer. The effects of such treatment on testicular function remained a concern for cases and clinicians.

**Objective:**

We aimed to observe changes in fertility indicators in men treated with ablation.

**Materials and Methods:**

In this prospective cohort study, 18 men with differentiated thyroid cancer from June to December 2020 underwent thyroidectomy plus radioiodine therapy. Participants were grouped based on iodine dose (8 men with 30 mCi vs. 10 men with ≥ 150 mCi). Baseline values (V ${}_{\text{B}}$ ) of the follicular stimulating hormone, luteinizing hormone, testosterone, and sperm analyses were measured 3 wk before iodine ablation and repeated 3 (V ${}_{3}$ ) and 12 (V ${}_{12}$ ) months later. They were analyzed once as a whole and once based on their groups via ANOVA and Friedman's tests where appropriate.

**Results:**

The mean age of participants was 35.61 ± 9.74 yr. Follicular stimulating hormone levels showed a significant trend among all participants (V ${}_{\text{B}}$ : 12.51 ± 1.72, V ${}_{3}$ : 13.54 ± 1.41, and V ${}_{12}$ : 13.10 ± 1.67 IU/mL; p < 0.001). Luteinizing hormone showed a similar pattern (V ${}_{\text{B}}$ : 4.98 ± 1.27, V ${}_{3}$ : 5.65 ± 1.29, and V ${}_{12}$ : 5.21 ± 0.95 IU/mL; p < 0.001). Testosterone levels did not differ significantly from baseline. Sperm count decreased at the first checkpoint and returned to normal after 12 months (V ${}_{\text{B}}$ : 38.22 ± 19.40, V ${}_{3}$ : 32.05 ± 17.96, and V ${}_{12}$ : 36.66 ± 18.81 million/mL; p < 0.001). Sperm motility and morphology did not change significantly.

**Conclusion:**

Our research showed that even less than 5 GBq irradiation could induce a transient testicular dysfunction in the first 3 months of therapy, but it was mostly reversible after 12 months.

## 1. Introduction

Thyroid cancer is the most common endocrine malignancy. In 2021, it accounted for 2.3% of all new cancer diagnoses (1, 2). The American Cancer Society estimated that 43,800 new cases would be diagnosed with thyroid cancer in 2022 (1). Differentiated thyroid cancer (DTC), namely follicular and papillary thyroid cancers, account for more than 90% of thyroid cancers. The standard management of DTC is surgical tumor removal followed by radioactive iodine (RAI) ablation (3). Iodine-131 (I-131) can harm the radiation-sensitive tissues, which are mostly composed of labile cells (cells that are continuously divided). Although multiple studies have focused on the effects of radiation on the female reproductive system, male fertility-related complications have not been considered widely (4-6). While women have their oocyte bank at birth in a quiescent status, spermatogonia undergo continuous meiosis and are more vulnerable to radiation.

The β and γ radiations from RAI therapy can cause testicular damage (4). Spermatogonia are amongst the most radiation-sensitive cells. The instant injury to the testes arises from direct radiation from I-131 in the blood and its accumulation in the bladder. Pelvic irradiation in the presence of pelvic metastases also directly affects spermatogonia. The released derivatives of irradiated thyroid or cancer cells, thyroglobulins, and thyroxine into the blood cause delayed harmful effects on spermatognia (3). The estimated absorptive dose of testis after RAI is 27-54 mGy/ giga-becquerel (GBq) (7, 8). DNA alterations are expected following irradiation. A case report showed that following a single dose of 150 millicuries (mCi)/5.56 GBq I-131 therapy, the DNA fragmentation index returned to normal values after 3.25 months following RAI (3). Thus, RAI ablation can affect sperm quality, and previous studies showed hormonal changes after such treatment modality (7, 9).

Thyroid cancer tends to affect individuals at a younger age compared to other adult malignancies. The median age at diagnosis was 51 yr, while 36.1% of cases occur at < 44 yr (2); thus, a delicate evaluation of testes function is important for men with DTC, especially for those candidates for high-dose RAI cases or who suffer pelvic metastases. Several studies have also recommended sperm banking before iodine therapy, which is still controversial (3, 10).

We aimed to observe the effects of iodine therapy on semen parameters and the hormonal status of men with DTC.

## 2. Materials and Methods

### Study design and participants

**Figure 1:**
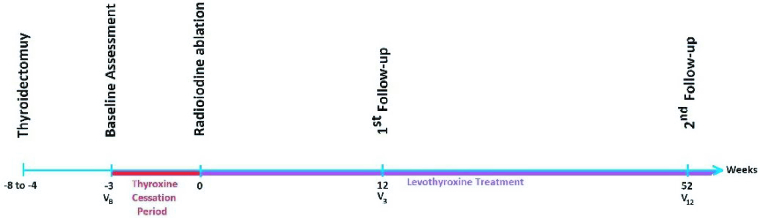
Chronology of events during the study.

This prospective cohort study evaluated the hormonal status and sperm analysis of men with well-differentiated thyroid carcinoma before and after iodine therapy. From June to December 2020, we included (in a census manner) all males with well-differentiated thyroid carcinoma aged between 20-45 yr, who were then referred to the Ghaem and Imam-Reza hospitals of Mashhad University Medical Sciences, Mashhad, Iran. Exclusion criteria were considered as not willing to participate, developing varicocele during the experiment, having previous cardiac, renal, metabolic, hepatic, and neurologic diseases, and having a history of any infertility intervention (medical and surgical). 18 men entered the study and were followed for 12 months after iodine therapy. Baseline assessment of the levels of luteinizing hormone (LH), follicle-stimulating hormone (FSH), testosterone, and sperm quality parameters (count, motility, and morphology) were done after thyroidectomy and before levothyroxine cessation, 3 wk before iodine therapy (Figure 1). Participants' sperm count, motility, and morphology were evaluated based on the 2010 World Health Organization criteria. We also interviewed participants for any history of sexual dysfunction, previous infertility, or related treatments. All men underwent a baseline urologic examination to assess confounding factors, like the development or progression of varicocele during the study.

### Ethical considerations

The Ethics Committee of Mashhad University of Medical Sciences, Mashhad, Iran, approved this study (Code: IR.MUMS.MEDICAL.REC.1399.277). All participants gave written informed consent for participation in the study and the anonymous publication of the results.

### Statistical analysis

We used the SPSS 24.0 software (IBM SPSS Statistics for Windows, Armonk, NY: IBM Corp.) to analyze the results with a p-value < 0.05 as the significance cut off. The included cases were analyzed once as a whole and once in groups. We used the Shapiro-Wilk test for normality assessment. Repeated measures ANOVA was used to analyze quantitative values with normal distribution. We also used Friedman's test to analyze variables lacking normal distribution.

## 3. Results

Out of 18 men who met the inclusion criteria, 8 cases received low-dose and 10 cases received high-dose iodine therapy. The mean ages were 37.62 ± 10.43, 34.00 ± 9.39, and 35.61 ± 9.74 yr for low-dose, high-dose, and total participants, respectively. Physical examination was normal in all cases except for 4 with clinical varicocele grades II-III (2 men in each group). None of the participants experienced infertility problems. The median cumulative iodine dose was 150 mCi (interquartile range: 30-150).

The mean levels of LH, FSH, testosterone, and sperm quality parameters (count, motility, and morphology) at baseline and 3 and 12 months after iodine therapy are presented in table I. The trend of these 3 checkpoints (repeated measures ANOVA or Friedman's test) is shown in the p-value column. During the study, significant changes were observed in all the participants LH and FSH values. However, the analysis revealed no statistically significant difference after grouping these men. Interestingly, the sperm count changes were significant with high-dose iodine therapy; however, it normalized after 12 months of RAI. Analysis of sperm motility by paired *t* test at baseline and after 12 months showed a significant decline (p = 0.043).

**Figure 2:**
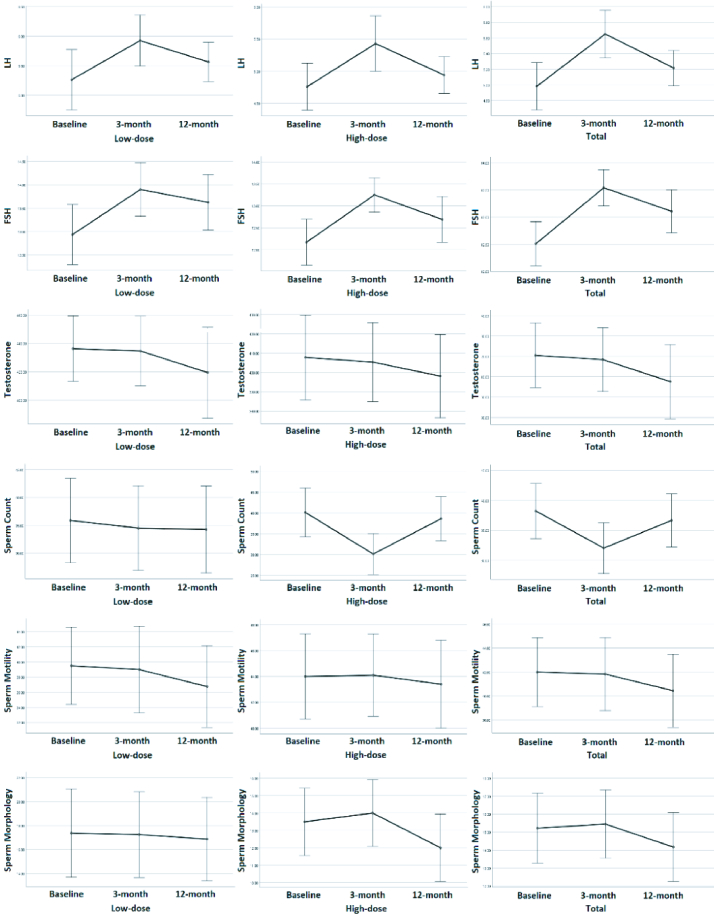
The trend for baseline measures and each checkpoint. LH: Luteinizing hormone, FSH: Follicular-stimulating hormone.

Figure 2 shows the trend for baseline measures and each checkpoint. The nadir and acme of each variable were unique chronologically; namely, the effects of iodine therapy were more significant in the 3-month follow-up for LH and FSH; however, its impact on morphology was more prominent at the 12-month follow-up.

**Table 1:** Fertility indicators and sperm analysis results at baseline, 3, and 12 months of iodine therapy


**Parameter**	**Iodine dose**	**Baseline**	**3 months**	**12 months**	**P-value**
	Low	5.26 ± 1.44	5.92 ± 1.22	5.56 ± 0.93	0.21
**LH (IU/mL)**	High	4.76 ± 1.15	5.43 ± 1.36	4.94 ± 0.91	0.10
	Total	4.98 ± 1.27	5.65 ± 1.29	5.21 ± 0.95	0.01
	Low	12.94 ± 1.81	13.90 ± 1.61	13.62 ± 1.67	0.08
**FSH (IU/mL)**	High	12.17 ± 1.66	13.25 ± 1.23	13.69 ± 1.65	0.06 ††
	Low	39.50 ± 14.46	39.00 ± 16.13	36.75 ± 15.25	0.03
**Sperm motility (%)**	High	44.00 ± 10.41	44.10 ± 10.08	43.40 ± 10.71	> 0.99 †† Friedman's test was used where data lacked normal distribution based on the Shapiro-Wilk test. LH: Luteinizing hormone, FSH: Follicular-stimulating hormone

## 4. Discussion

Our study aimed to evaluate the effects of iodine therapy on spermogram parameters in men with DTC. After 3 months, a significant increase in FSH and LH levels was detected at the first checkpoint, which returned to baseline at the last checkpoint. We also detected a significant decrease in sperm count, whereas no significant changes were observed in sperm motility and morphology.

RAI ablative therapy in managing DTC improves outcomes by targeting potential micrometastases. The treatment's main adverse effects (sialadenitis, gastrointestinal discomfort, leukemia, etc.) mostly involve rapid-cycling cells with many divisions (8). The germinal epithelium of the testis also consists of labile cells, and several studies have evaluated the effects of iodine therapy on sperm parameters (5, 6, 10, 11). Even an absorptive dose of 0.1 Gy can temporarily impair spermatogenesis (11). We detected a significant increase in FSH levels at the first checkpoint after 3 months. Although the changes seemed temporary, these measures tend to return to baseline at the last checkpoint. Multiple studies affirm a transient increase in FSH levels following iodine therapy; FSH tended to decrease toward normal levels after 9-12 months of RIA (9, 10). No statistical significance was observed in our subgroup analysis between low-dose (p = 0.081) and high-dose (p = 0.061) RAI in the magnitude of increased FSH levels. One has to interpret these results with caution, as several experiments evaluating higher radiation doses have declared a dose-dependent relation between RAI and FSH levels. Higher doses of RAI cause a more profound increase in FSH levels, but it is negligible as it mostly comes back toward normal levels (9, 10). Persistent FSH elevation was seen in cumulative doses higher than 14 GBq/378 mCi (10). However, another study with 25 participants did not show a dose-dependent effect on FSH levels; it should be noted that they compared radiation doses of 150-300 mCi with doses higher than 300 mCi (7, 8). A recent long-term study showed that RAI doses of 100 mCi/3.7 GBq or higher did not affect reproductive function, and sperm banking was not necessary routinely (4). However, men who desire to conceive within the first year of DTC ablation therapy should be informed about sperm quality and hormonal alterations after ablation.

LH and testosterone and the testosterone/LH ratio are indicators of Leydig cell function or testicular endocrine function (10). No significant differences were observed in testerone levels when LH levels were mirrored with the FSH changes. Such a trend has been observed in multiple studies (9, 10). Several studies similar to these surveys showed a significant increase in LH levels (from 3.0 ± 0.2 to 4.2 ± 0.3) with no changes in testosterone levels, suggesting a transient disturbance in Leydig cell function (9, 10). On the contrary, another study detected a significant LH increase in 9.6% of 52 cases who received 3.7-5.5 GBq radiation, while 22.7% of 22 cases who received ≥ 13 GBq radiation dose had elevated LH levels (10, 12). Another possible explanation for mentioned controversy on LH pattern is that Leydig cells are affected more than Sertoli cells in hypothyroidism (13); thus, individuals' adherence to thyroxine hormone replacement can potentially cause an impediment in LH trend assessment. As free testosterone remains in the normal range after RAI therapy, the exocrine functions of the testis seem to be more affected than endocrine roles (7). The impairment of testosterone production is expected at absorptive radiation doses greater than 20 Gy as the Leydig cells are more resistant to radiation (11). Future studies should focus on the exact effect of RAI on LH and testosterone levels.

We also observed changes in sperm count, motility, and morphology, of which sperm count changed significantly with an initial decrease followed by returning to normal after 12 months. However, sperm motility and morphology started to decline in the 12 ${}^{\text{th}}$ month. Bourcigaux et al. found similar sperm concentration and motility results, but sperm morphology changes began after 3 months (10). In terms of long-term assessment, a recent study evaluated 51 men with DTC after a median of 5.8 yr (interquartile range: 3.0-9.5) after their last dose of I-131 with a cumulative dose of ≥ 3.7 GBq/100 mCi (4). They concluded that sperm count, progressive motility, and volume did not differ significantly from the general population and advised that semen banking should be considered individually. More extended damage is expected where azoospermia occurs with absorptive doses of 4 Gy (11).

The timing of hormonal measurement is crucial in assessing the fertility status of individuals with DTC treated with RAI. The TSH levels should be over 25 to ensure sufficient iodine absorption into the thyroid tissue(s); consequently, measuring FSH and LH when TSH is this high probably gives lower than expected results (14). We found one experiment that appropriately addressed this issue and performed hormonal analysis immediately before levothyroxine withdrawal (10), but some others have measured FSH and LH levels during the thyroxine withdrawal period (7, 8). Another finding of our observation was that the nadir and acme of each hormone occur at a specific checkpoint; as explained, iodine therapy affected LH and FSH at the 3-month follow-up. However, its impact on morphology was more prominent during the 12-month follow-up. Although it is transient, a thorough consultation with cases regarding fertility issues is needed if a patient desires to conceive within the first year of RAI therapy. It has also been shown previously for women that the risk of abortion increases within the first year of treatment (15).

### Limitation and suggestions

The most significant limitation of the present study was its small sample size, probably secondary to not considering the psychological burden of the diagnosis on cases. Several cases refrained from participating because they did not receive proper psychological support after the cancer diagnosis, and asking them to participate in a study was relatively premature. Although this small sample size can compromise the validity of our conclusion, as it is mostly in line with similar studies of the female population (in terms of LH and FSH), we believe this effect is negligible. Other limitations were not including TSH, prolactin, inhibin B, and DNA fragmentation index in our fertility analysis. We suggest further studies in performing baseline hormonal assessment before the cessation of thyroid hormones for RAI therapy. Assessing baseline and follow-up of TSH levels helps determine individuals' adherence to thyroid hormone replacement and provides more reliable results. Due to insufficient data on the effects of iodine therapy on prolactin, evaluation of this hormone could be considered. Occupational status, previous history of medications, smoking, body mass index, and probable fever episodes in the last 3 months should also be considered.

## 5. Conclusion

Low-dose iodine therapy in men with DTC can cause short-term spermatogenesis defects, which are usually relieved within a year. Higher doses expectedly cause more profound damage, especially on sperm count. Men experience increased FSH and LH levels with no significant change in testosterone. Sperm count is affected more than sperm motility or morphology during the first year. Although such effects are transient, a thorough consultation regarding fertility issues is needed if a man desires to conceive within the first year of radioiodine treatment.

##  Conflict of Interest

The authors declare that there is no conflict of interest.

## References

[B1] National Cancer Institute

[B2] National Cancer Institute

[B3] Esquerré-Lamare C, Isus F, Moinard N, Bujan L (2015). Sperm DNA fragmentation after radioiodine treatment for differentiated thyroid cancer. Basic Clin Androl.

[B4] Nies M, Arts EGJM, van Velsen EF, Burgerhof JGM, Kobold ACM, Corssmit EPM, et al (2021). Long-term male fertility after treatment with radioactive iodine for differentiated thyroid carcinoma. Eur J Endocrinol.

[B5] van Velsen EFS, Visser WE, van den Berg SA, Kam BL, van Ginhoven TM, Massolt ET, et al (2020). Longitudinal analysis of the effect of radioiodine therapy on ovarian reserve in females with differentiated thyroid cancer. Thyroid.

[B6] Yaish I, Azem F, Gutfeld O, Silman Z, Serebro M, Sharon O, et al (2018). A single radioactive iodine treatment has a deleterious effect on ovarian reserve in women with thyroid cancer: Results of a prospective pilot study. Thyroid.

[B7] Wichers M, Benz E, Palmedo H, Biersack HJ, Grünwald F, Klingmüller D (2000). Testicular function after radioiodine therapy for thyroid carcinoma. Eur J Nucl Med.

[B8] Fard-Esfahani A, Emami-Ardekani A, Fallahi B, Fard-Esfahani P, Beiki D, Hassanzadeh-Rad A, et al (2014). Adverse effects of radioactive iodine-131 treatment for differentiated thyroid carcinoma. Nucl Med Commun.

[B9] Canale D, Ceccarelli C, Caglieresi C, Moscatelli A, Gavioli S, Santini P, et al (2015). Effects of radioiodine treatment for differentiated thyroid cancer on testis function. Clin Endocrinol.

[B10] Bourcigaux N, Rubino C, Berthaud I, Toubert ME, Donadille B, Leenhardt L, et al (2018). Impact on testicular function of a single ablative activity of 3. 7 GBq radioactive iodine for differentiated thyroid carcinoma Hum Reprod.

[B11] Sayan M, Cassidy R, Butker EE, Nanda RH, Krishnamurti L, Khan MK, et al (2016). Gonadal shielding technique to preserve fertility in male pediatric patients treated with total body irradiation for stem cell transplantation. Bone Marrow Transplant.

[B12] Rosario PW, Xavier ACM (2012). Recombinant human thyroid stimulating hormone in thyroid remnant ablation with 1. 1 GBq 131iodine in low-risk patients Am J Clin Oncol.

[B13] Ambigapathy JS, Kamalanathan S, Sahoo J, Kumar R, Perumal NL (2020). Effect of thyroxine replacement on leydig cell and sertoli cell function in men with hypothyroidism. Indian J Endocrinol Metab.

[B14] Alahmar A, Dutta S, Sengupta P (2019). Thyroid hormones in male reproduction and infertility. Asian Pac J Reprod.

[B15] Zhang L, Huang Y, Zheng Y, Cai L, Wen J, Chen G (2021). The effect of I-131 therapy on pregnancy outcomes after thyroidectomy in patients with differentiated thyroid carcinoma: A meta-analysis. Endocrine.

